# Copy Number Variations of Four Y-Linked Genes in Swamp Buffaloes

**DOI:** 10.3390/ani10010031

**Published:** 2019-12-22

**Authors:** Ting Sun, Quratulain Hanif, Hong Chen, Chuzhao Lei, Ruihua Dang

**Affiliations:** 1 Key Laboratory of Animal Genetics, Breeding and Reproduction of Shaanxi Province, College of Animal Science and Technology, Northwest A&F University, Yangling 712100, Shaanxi, China; 2 National Institute for Biotechnology and Genetic Engineering, Pakistan Institute of Engineering and Applied Sciences, Faisalabad 577, Pakistan

**Keywords:** swamp buffalo, CNVs, Y-linked genes

## Abstract

**Simple Summary:**

The amplification of the male-specific region of the Y chromosome was a unique phenomenon during mammalian sex chromosome evolution. The Y-linked copy number variations of many species have been confirmed. However, the Y-linked copy number variations (CNVs) in water buffalo are still unknown. In this study, we investigated the copy number variations of four Y-linked genes (*SRY*, *UTY*, *DBY*, and *OFD1Y*) in buffalo. Our results showed that *UTY* was a single-copy gene in buffalo, while *DBY*, *OFD1Y*, and *SRY* exhibited copy number variations in buffalo.

**Abstract:**

Copy number variation (CNV), a significant source of genetic diversity in the mammalian Y chromosome, is associated with the development of many complex phenotypes, such as spermatogenesis and male fertility. The contribution of Y-linked CNVs has been studied in various species, however, water buffalo has not been explored in this area and the genetic information still remains unknown. The aim of the current study was to investigate the CNVs of four Y-linked genes, including, *sex determining Region of Y-Chromosome* (*SRY*), *ubiquitously transcribed tetratricopeptide repeat gene protein on the chromosome Y* (*UTY*), *DEAD-box helicase 3 Y-linked (DDX3Y*, also known as *DBY*), and *oral-facial-digital syndrome 1 Y-linked* (*OFD1Y*) in 254 swamp buffaloes from 15 populations distributed across China, Vietnam, and Laos using quantitative real-time PCR (qPCR). Our results revealed the prevalence of a single-copy *UTY* gene in buffaloes. The *DBY* and *OFD1Y* represented CNVs among and within different buffalo breeds. The *SRY* showed CNVs only in Vietnamese and Laotian buffaloes. In conclusion, this study indicated that *DBY*, *OFD1Y*, and *SRY* showed CNVs, while the *UTY* was a single-copy gene in swamp buffaloes.

## 1. Introduction

Copy number variation (CNV) is a significant source of genetic diversity, comprised of varying copy numbers of DNA segments (at least 50 bp in size) within species [1]. CNVs are known to be associated with gene expression, phenotypic variation, adaption, and the development of various diseases [2,3]. In cattle, the CNV is associated with resistance or susceptibility to gastrointestinal nematodes [4], production traits [5,6], and muscle [7]. Studies showed that the CNV also plays a key role in the coat color in goat [8], pig [9], and horse [10].

The mammalian Y chromosome stands out from rest of the genome because it is constitutively haploid and escapes recombination for most of its length, leading to a correspondingly unusual genomic landscape [11]. Chromosome Y is rich in segmental duplications and provides a rich environment for the generation of CNVs [11,12,13]. The male-specific region of the Y chromosome (MSY) accounts for 95% of the Y chromosome, consisting of three regions: ampliconic, X-transposed, and X-degenerate. The MSY is enriched with multi-copied genes and contains different and some specific MSY genes in different species [11,14,15,16,17].

Water buffalo (*Bubalus bubalis*), an important livestock in tropical and subtropical climates, can provide meat, milk, and hides for people [18]. Water buffalo can be divided into two types according to body size, outward appearance, biological characteristics, and chromosome karyotype: swamp (2n = 48) and river buffalo (2n = 50) [19,20]. The CNVs of river buffalo on the autosomal and X chromosomal have been investigated [21,22]. However, there are few studies about the Y-linked CNV genes in buffalo. Only Zhang et al. [23] detected the CNVs in two multicopy-Y-linked genes (*HSFY* and *ZNF280BY*) in buffaloes, where swamp buffalo showed significant CNV abundance. To date, there is no research about CNVs of X-degenerate genes in buffaloes. In the current study, four X-degenerate genes, *sex determining Region of Y-Chromosome* (*SRY*), *ubiquitously transcribed tetratricopeptide repeat gene protein on the chromosome Y* (*UTY*), *DEAD-box helicase 3 Y-linked (DDX3Y*, also known as *DBY*), and *oral-facial-digital syndrome 1Y-linked* (*OFD1Y*), were selected to determine their copy numbers on buffalo Y chromosome. *SRY* gene, mainly associated with sex differentiation and male sexual development, was used as a candidate marker for sperm quality and fertility in bulls [24]. Studies showed that *SRY* is present as a single-copy gene in human, mouse, chimpanzee, horse, and cattle [14,16,25,26], while as a multi-copy gene in domestic cat and rabbit [27,28]. *DBY* and *UTY* are known to play key roles in spermatogenesis and bull fertility [29,30]. *DBY* and *UTY* were also identified as single copy genes in human, chimpanzee, horse, cattle, and mouse [14,16,25,26]. *OFD1Y* may play an important role in sperm development, which is associated with infertility in cattle [31], and highly expressed in porcine testis [32]. *OFD1Y* is a single-copy gene in cattle whose function is still unknown [16,33,34], but there are at least 14 and 18 copies in chimpanzee and human, respectively [33].

In our previous studies, several mutation sites in *SRY*, *DBY*, and *OFD1Y* genes were identified [35]. Then, the authors chose one sample to perform the TA cloning for further verification. Finally, 40 cloning sequences were obtained in *SRY*, *DBY*, and *OFD1Y* genes of the sample, respectively, and showed several mutation sites. These genes were located in the MSY region which is a non-recombining region with no X–Y crossing over [11], but single nucleotide polymorphisms (SNPs) were in the sample [35]. So, the authors deduced that *SRY*, *DBY*, and *OFD1Y* were multi-copy genes in buffaloes [35]. However, their CNVs were unknown. The aim of the study was to investigate the CNVs of *SRY*, *DBY*, *OFD1Y*, and *UTY* of two river buffaloes and 254 swamp buffaloes from 15 populations distributed in China, Vietnam, and Laos using qPCR.

## 2. Materials and Methods 

In total, 254 male buffalo samples (including 204 ear tissues and 50 blood samples) were collected from 15 swamp buffalo populations (including 254 swamp buffaloes) in China, Vietnam, and Laos. The study was approved by the Institutional Animal Care and Use Committee of Northwest A&F University. The genomic DNA was extracted from the ear tissues or the blood using a standard phenol-chloroform method [36]. PCR primers were designed using the Primer Premier 5.0 (Table S1). *Basic transcription factor 3* (*BTF3*; XM_006062116), a two-copied gene in bovine, was chosen as a reference gene [37,38].

To validate the male specificity of primers used in this study, a routine PCR was performed. Five female buffaloes were used as negative controls, and water was used as a blank control. The PCR protocol was as follows: each 12.5 μL reaction contained 1 μL of genome DNA (10 ng/μL), 0.5 μL of each primer (10 pmol/μL), 6.25 μL of 2 × PCR Mix buffer (CWBIO), and 4.25 μL of distilled water. Thermocycling consisted of an initial denaturation at 95 °C for 5 min, followed by 35 cycles at 94 °C for 30 s, annealing temperature (Table S1) for 40 s, and 72 °C for 30 s, followed by a final extension at 72 °C for 10 min. The PCR products were visualized on 1% agarose gel electrophoresis.

qPCR was used to measure the *SRY*, *DBY*, *UTY*, and *OFD1Y* copy numbers using the Roche LightCycler 480. Standard curves were generated from male buffalo DNA diluted to 40, 20, 10, 5, 2.5, 1.25, and 0.625 ng/μL for *SRY*, *DBY*, *UTY*, and *OFD1Y*. Primer efficiencies were calculated based on the slopes of standard curves and the equation E = 10 ^[1/slope]^ [39]. For the test samples, DNA was concentrated to 5 ng/μL. The qPCR reaction contained 5 μL of SYBR Green PCR Master Mix (TAKARA), 0.5 μL of primers (10 pmol/μL), 3 μL of distilled water, and 1 μL of DNA template. Each sample and calibrator were run in triplicate using a standard shuttle PCR protocol (pre-denaturation at 95 °C for 30 s followed by of denaturation at 95 °C for 5 s and annealing at 60 °C for 20 s for 40 cycles). A melting curve was then generated by taking fluorescent measurements every 0.1 °C from 65–95 °C.

The DNA sample of a Yibin buffalo was used to calibrate cycle threshold (C_T_) variations among the plates. The C_T_ value of the calibrator for each gene was determined using average C_T_ values of all the plates for the corresponding sample. The copy numbers were estimated using the following three equations:

(1) $$\mathrm{Copy}\;\mathrm{number}\;\mathrm{calibrator}\;=\;\frac{{\left({E\;}_{\mathrm{reference}}\right)}^{C_{T\;\mathrm{reference}}}}{{\left({E\;}_{\mathrm{target}}\right)}^{C_{T\;\mathrm{target}}}}$$

(2) $$\mathrm{Ratio}\;=\;\frac{{\left({E\;}_{\mathrm{target}}\right)}^{\;\Delta C_{T\;\mathrm{target}}\;\left(\mathrm{calibrator}\;-\;\mathrm{sample}\right)}}{{\left({E\;}_{\mathrm{reference}}\right)}^{\;\Delta C_{T\;\mathrm{reference}}\;\left(\mathrm{calibrator}\;-\;\mathrm{sample}\right)}}$$

Copy number test sample = (Copy number _calibrator_) × (ratio) × 2.

In order to minimize technical errors and to have accurate copy number estimates, 11 samples with high variation in raw C_T_ values (CV >1%) among the triplicates were excluded from further analysis.

To verify the accuracy of qPCR results, TA cloning was performed for these four genes with the samples exhibiting different copy numbers from the others. PCR products were purified using Universal DNA Purification Kit (TIANGEN, Beijing, China), then ligated into the cloning vector pGEM-T Easy and transformed into *Escherichia coli* DH-5α (CWBio, Beijing, China). Then, 10–15 clones per sample were picked and amplified by PCR method. PCR products were sequenced on an ABI PRIZM 377 DNA sequencer (Perkin-Elmer, Foster City, CA, USA) (Shanghai Sangon Biotech Company, Shanghai, China).

## 3. Results and Discussion

**Figure 1:**
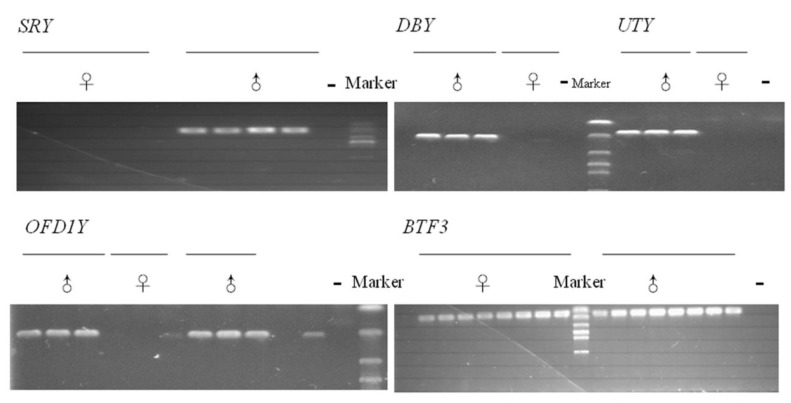
Gel electrophoresis of PCR products of the buffalo *sex determining Region of Y-Chromosome* (*SRY*), *ubiquitously transcribed tetratricopeptide repeat gene protein on the chromosome Y* (*UTY*), *DEAD -box* helicase 3 Y-linked (DDX3Y, also known as *DBY*), and *oral-facial-digital syndrome 1 Y-linked* (*OFD1Y*) genes. The primers of the SRY, DBY, UTY, and OFD1Y genes amplified male-specific band. Marker, D2000 DNA ladder; the “♂” represents the male buffalo genomic DNA; the “♀” represents the female buffalo genomic DNA; the “-” represents the blank control (distilled water).

The results showed that each pair of primers amplified a male specific band with the expected fragment size, confirming that the designed primers were male-specific and could be used for the qPCR analysis in this study (Figure 1). The primer efficiency of *SRY*, *DBY*, *UTY*, and *OFD1Y* were 1.98, 1.96, 2.07, and 2.02, respectively (Table S1).

Our results showed that the median copy numbers (MCNs) of *SRY*, *DBY*, *OFD1Y*, and *UTY* were 1, 2, 1, and 1 with CNVs ranges of 1–4, 1–8, 1–4, and 1, respectively (Table 1). The MCN and copy number of *SRY* gene was 1 in 13 Chinese native buffalo populations. Yet the MCN was 2 with the CNVs ranging from 1 to 4 in Laotian and Vietnamese buffalo, indicating that the *SRY* is a multi-copy gene in buffalo. The MCN of *DBY* was 2 with its copy number ranging from 1 to 8. Thus, we defined that *DBY* is a multi-copy gene in buffalo. The MCN and copy number of *DBY* is variable among individuals and populations. The Fuling population possessed the highest MCN (6), while the Guangxi and Yibin populations exhibited the lowest MCN (1). The MCN and the copy number of *UTY* gene was 1 in all tested buffaloes, which indicated that *UTY* was a single-copy gene in buffalo. The MCN of *OFD1Y* gene was 1 with the copy number ranging from 1 to 4 in several populations. So, we concluded that *OFD1Y* is a multi-copy gene in buffalo populations.

The results of qPCR indicated that *SRY*, *DBY*, and *OFD1Y* showed CNVs within buffalo populations, which were in accordance with our previous cloning results [35]. In different species, the copy number of the Y-linked genes may be different [15]. *SRY*, *DBY*, and *UTY* act as single-copy genes in human, mouse, chimpanzee, horse, and cattle [14,16,25,26], while *UTY* is a multi-copy gene in domestic cat and rabbit [33]. *OFD1Y* showed copy number variations in chimpanzee and human, but demonstrated to be a single-copy gene in cattle [16,33,34]. Studies also showed that the copy number of genes varied from individual to individual [11]. The male-specific region is enriched in multi-copy gene families that are all predominantly expressed, or solely in testis, and involved in spermatogenesis and male fertility [11,16,39,40]. Previous studies have demonstrated that the Y-linked CNVs are associated with male reproductive traits [39], testis size, and bull fertility in cattle [40]. Although CNVs of the Y-linked gene families and their function have not been well-studied in buffalo due to the unavailability of the Y chromosome sequences, we boldly speculate that these four genes may also play an important role in the spermatogenesis and male fertility in buffaloes; yet, more work should be done to verify this corollary in future.

## 4. Conclusions

In conclusion, we found that the buffalo *UTY* gene was a single-copy gene on the Y chromosome, while the copy numbers of *SRY*, *DBY*, and *OFD1Y* were variable among buffalo populations. These three genes (*SRY*, *DBY*, and *OFD1Y*) demonstrated large-scale CNVs among individuals and populations, which provide great genetic resources for assessing their association with male fertility in future work.

## Figures and Tables

**Table 1:** The median copy number of *SRY*, *DBY*, *UTY*, and *OFD1Y* genes in 16 buffalo populations.

			Median Copy Number			
Population	Abbreviation	Sample Size	*SRY*	*DBY*	*UTY*	*OFD1Y*
Dehong	DH	20	1	2 (1–4)	1	1
Diandongnan	DDN	7	1	2 (1–2)	1	1 (1–2)
Guizhou	GZ	4	1	2 (1–2)	1	1
Guangxi	GX	11	1	1 (1–2)	1	1
Fuling	FL	7	1	6 (2–8)	1	1
Xinyang	XY	33	1	2 (1–3)	1	1 (1–4)
Jianghan	JH	14	1	3 (1–4)	1	1 (1–2)
Guangyuan	GY	27	1	2 (1–3)	1	1
Yibin	YB	28	1	1 (1–2)	1	1 (1–2)
Haizi	HZ	4	1	2 (1–3)	1	1
Xiajiang	XJ	25	1	2 (1–4)	1	1
Poyanghu	PYH	24	1	2 (2–3)	1	1 (1–2)
Jianghuai	JHU	14	1	2 (1–3)	1	1 (1–2)
Vietnamese	VN	26	2 (1–4)	2 (1–4)	1	2 (1–4)
Laotian	LA	12	2 (1–4)	2 (1–4)	1	1 (1–2)
Total		254	1	2	1	1

Note: The numbers in the “()” represent the range of copy numbers of *SRY*, *DBY*, *UTY*, and *OFD1Y* genes.

## Supplementary Materials

The following are available online at https://www.mdpi.com/2076-2615/10/1/31/s1. Table S1: The information of primers used in qPCR.
