# Supplementary material for: Copy Number Variations of Four Y-Linked Genes in Swamp Buffaloes
Source: Animals (Basel). 2019 Dec 22;10(1):31. doi: 10.3390/ani10010031 (PMC7023270; doi:10.3390/ani10010031)
Supplement: Supplementary file 1 [file animals-10-00031-s001.pdf]

animals

Supplementary

Table S1. The information of primers used in qPCR

Primer name	Sequence	Annealing (°C)	Tm Primer efficiency	Size (bp)
BTF3 F	5′-AACCAGGAGAAACTCGCCAA-3′	64	1.98 at 64°C; 1.95 at 63°C; 2.02 at 62°C	166
BTF3 R	5′-TTCGGTGAAATGCCCTCTCG-3′			
SRY F	5′-GATTTATTTGGGAGAAGGCTTC-3′	62	1.98	242
SRY R	5′-GTGGGGTTGGTGTTTTGTGTC-3′			
DBY F	5′-AGCAAAGCACAGAGGGGAG-3′	62	1.96	250
DBY R	5′-GTCAAGAGGAAGACACAGTTAC-3′			
UTY F	5′-GGCGTCTTAGTCACTTTATCACAT-3′	62	2.07	193
UTY R	5′-CAAGGGACTGAGGGACATAAGC-3′			
OFD1Y F	5′-CGACATCTCAATGAAGAAAGGG-3′	62	2.02	231
OFD1Y R	5′-CTCACTCTGTGTGTGTTTGCTC-3′			

© 2019 by the authors. Submitted for possible open access publication under the terms and conditions of the Creative Commons Attribution (CC BY) license (http://creativecommons.org/licenses/by/4.0/).
